# Circulating Profile of ECM-Related Proteins as Diagnostic Markers in Inflammatory Bowel Diseases

**DOI:** 10.3390/jcm11195618

**Published:** 2022-09-23

**Authors:** Katarzyna Komosinska-Vassev, Aleksandra Kałużna, Agnieszka Jura-Półtorak, Alicja Derkacz, Krystyna Olczyk

**Affiliations:** Department of Clinical Chemistry and Laboratory Diagnostics, Faculty of Pharmaceutical Sciences in Sosnowiec, Medical University of Silesia in Katowice, 41-200 Sosnowiec, Poland

**Keywords:** inflammatory bowel diseases, ECM remodeling, laminin, fibronectin, neutrophil gelatinase-associated lipocalin

## Abstract

The aim of our research was to find new biomarkers that could be potentially used in the diagnosis, differentiation and monitoring of inflammatory bowel diseases (IBD). Since extracellular matrix (ECM) remodeling contributes to the pathological changes occurring in IBD, the serum profile of ECM-related proteins may reflect disease activity in the intestinal mucosa. Serum laminin (LM), fibronectin (FN) and gelatinase-associated lipocalin (NGAL) concentrations were determined in 51 patients with IBD before and after a year of treatment, as well as in 48 healthy individuals. A significant difference in serum concentration of FN (130,56 ± 52.87 vs. 287.93 ± 79.69, *p* < 0.001) and NGAL (133.34 ± 51.51 vs. 102.37.39, *p* < 0.05) between patients with ulcerative colitis (UC) and healthy individuals was found. In patients with Crohn’s disease (CD), serum concentrations of LM (1329.5 ± 389.36 vs. 1012.07 ± 260.85, *p* < 0.005) and NGAL (138.94 ± 51.31 vs. 102.65 ± 37.39, *p* < 0.05) were increased, while FN (89.26 ± 43.86 vs. 287.93 ± 79.69, *p* < 0.001) was decreased compared to healthy subjects. Moreover, a significant correlation was found between the Mayo score in patients with UC and the levels of NGAL (r = 0.49, *p* < 0.01) and LM (r = 0.035, *p* < 0.005), respectively. Another significant correlation was noted between the Crohn’s Disease Activity Index (CDAI) and LM (r = 0.49, *p* < 0.05) levels in CD group. The results presented in our studies indicate that ECM-related markers might be potential additional tools helpful in diagnosing IBD, differential diagnosis of UC and CD and monitoring the disease activity.

## 1. Introduction

Inflammatory bowel diseases include two main types: ulcerative colitis and Crohn’s disease, which are characterized by chronic inflammation developing in the gut. Diagnosing IBD and further differentiating it into ulcerative colitis (UC) or Crohn’s disease (CD) is a serious challenge. The most frequently used diagnostic tool is endoscopic examination, which is valuable, although very invasive, examination. Therefore, there is a serious need to find new, reliable and non-invasive methods, which would allow the diagnosis, monitoring and prediction of the course of disease [1,2].

One of the potential new biomarkers might be that which is related to the extracellular matrix (ECM), since ECM remodeling has been known to play a crucial role in the pathogenesis of IBD [3,4,5,6]. Inflammation of the gut, which is an integral part of the IBD process, disrupts the balance between ECM degradation and deposition, which is dependent on the activity of protease enzymes and the synthesis of ECM components. Increased expression of matrix metalloproteinases (MMPs) or other proteases (meprins, NE-neutrophil elastase) contributes to the destruction of the intestinal tissue and to the release of ECM’s components into the circulation. Moreover, during intestinal chronic inflammation, pro-inflammatory and profibrotic mediators lead to permanent activation of myofibroblasts (MFs), which shifts the processes of tissue repair toward tissue fibrosis as a result of excessive matrix deposition [1,7]. Since the released components of the ECM turnover reflect metabolic changes at the tissue level, collagens and glycosaminoglycans have been investigated as new diagnostic markers in IBD [8,9]. Surprisingly however, little is known about the diagnostic usefulness of other components of the ECM, including non-collagen ECM proteins such as laminin and fibronectin.

As the laminin is the most abundant non-collagenous protein of the basement membrane (BM), it may play a crucial role in the development of IBD. LM as a component of BMs plays a critical role in organizing the complex interactions with other ECM proteins including syndecan, nidogen, type VII collagen and integrins. Via cell receptors such as integrins or syndecans, laminins are able to regulate cell proliferation, migration and adhesion [4,7]. Most importantly, LM has been found to stimulate the release of neutrophil extracellular traps (NETs) by neutrophils, which are recruited from the blood and transmigrate through an LM layer in the vascular BM to reach the inflammatory site in response to inflammation.

Laminin shares several properties with fibronectin, another component of ECM with possible contribution to the development of IBD. FN is known to affect the cellular behavior by promoting fibroblast migration and proliferation acting mostly indirectly, by the release of TGF-β. Moreover, fibronectin polymerization promotes proper collagen fibrillogenesis and stabilizes the ECM during healing [10,11,12]. In IBD, the inflammatory processes trigger constant regeneration of ECM leading to tissue fibrosis and release of ECM components, including FN, to the surrounding tissues and then to the vessels. Only a few studies have presented a decrease in plasma FN concentration in patients with UC and CD compared to healthy subjects [13,14,15].

Another potential biomarker associated with both inflammation during IBD and ECM remodeling is neutrophil gelatinase-associated lipocalin. NGAL, also named lipocalin-2, is considered to play a pro-inflammatory role as it is released from neutrophil grains upon their activation and seems to fill a chemoattractant role for neutrophils [6,16,17]. Furthermore, NGAL takes part in ECM remodeling since it binds with MMP-9 and as a result, it stabilizes the enzyme and protects it from inactivation by tissue inhibitor of metalloproteinase-1 (TIMP-1). NGAL/MMP-9 complex prolong proteolytic activity of MMP-9 towards ECM. Observation of IBD excessive activation in immune cells results in enhanced release of pro-inflammatory cytokines (such as TNF-α, IL-1) that activate NF-κB pathway. The activation of the mentioned pro-inflammatory pathway leads to an increase in NGAL, as well as MMP-9 expression, indicating a key role of the inflammatory process in ECM remodeling [6,17,18].

In this study, we evaluated the potential usefulness of serum NGAL, laminin and fibronectin levels as ECM-related biomarkers potentially helpful in the diagnostic, differential diagnosis or therapy monitoring in patients with UC or CD.

## 2. Materials and Methods

### 2.1. Study Population

The material investigated in the research was venous blood collected from healthy controls and patients with inflammatory bowel diseases, including patients with Crohn’s disease and ulcerative colitis. The diagnosis of CD and UC was made at the Department of Gastroenterology of St. Barbara’s Regional Specialist Hospital in Sosnowiec, on the basis of clinical symptoms, endoscopic examination and laboratory tests. In addition, disease activity was assessed using the Mayo scale in patients with UC and the Crohn’s Disease Activity Index in CD patients.

### 2.2. Patients with Ulcerative Colitis—Inclusion and Exclusion Criteria

Patients involved in this study included a group of subjects selected for the program of treatment with Adalimumab. The inclusion criteria for patients with ulcerative colitis included an age between 18 and 75, an active form of ulcerative colitis diagnosis and confirmed through colonoscopy with biopsy, an active form of ulcerative colitis confirmed through the Patient Global Assessment (PGA) 2 or 3, previous treatment with corticosteroids or immunosuppressants azathioprine (AZA) or 6-mercaptopurine (6-MP) that had not brought about the results intended in the practitioner’s opinion, participation in concurrent treatment employing at least one of the following (oral corticosteroids, immunosuppressants, or both, as defined below): oral corticosteroids (prednisone ≥ 20 mg/day) for at least 14 days before randomization, or oral corticosteroids (prednisone < 20 mg/day) for at least 21 days before the randomization and/or at least 12 consecutive weeks (84 days) of AZA or 6 MP treatment before the post-randomisation examination. In the case of patients who were taking glucocorticosteroids and were included in the Adalimumab clinical treatment program, dosage was reduced starting from the 4th week of treatment.

Adalimumab doses delivered to patients with UC varied and the patients were randomly assigned to one of two groups: the first one received 160 mg of adalimumab in the initial dose, with a reduction to 80 mg and then to 40 mg in these patients there was a need for a faster response to treatment; the second group received 160 mg in the initial dose, with a reduction to 40 mg.

The exclusion criteria in the case of patients with UC included unstable coronary disease, chronic liver disease, chronic kidney disease, fulminant or toxic colitis, colectomy with ileorectostomy or colectomy with anastomosis, pregnancy or breastfeeding. Other exclusion criteria in the research were biological treatment with other preparations, rectal therapy with simultaneous infusions, receiving live vaccines and prior planned bowel surgery.

### 2.3. Patients with Crohn’s Disease—Inclusion and Exclusion Criteria

The second investigated group of patients included adult individuals diagnosed with Crohn’s disease. Patients with CD were new patients, who were not treated prior to our study. All the patients selected for the research program were treated with the prednisone, for which dosage was determined for each patient individually.

The exclusion criteria among patients with CD included chronic liver or kidney disease, unstable coronary disease, pregnancy or breastfeeding, diabetes, severe viral, fungal or bacterial infections, mild or acute myocardial failure, chronic respiratory failure, precancerous or cancerous conditions.

### 2.4. Control Subjects

The reference material for this study was venous blood drawn from adult healthy subjects. The subjects enrolled into the control group had not undergone surgical procedures within the previous 12 months, had not been hospitalized and they had not undergone pharmacological treatment. Subjects were also excluded if they took vitamin antioxidants, steroidal and non-steroidal anti-inflammatory drugs or if they had an infection at least 4 weeks prior to investigation. Patients enrolled into this study had morphological and biochemical analyses (transaminases, bilirubin, creatinine, lipid profile, total protein, C-reactive protein (CRP) and fasting glucose within the reference range. In addition, patients were not overweight and had BMI < 25 kg/m^2^ and their blood pressure profile was in the normal range.

### 2.5. Biological Material for Research

Biological material used in this research was venous blood collected from the elbow vein to the test tubes with no added anticoagulant. The blood was centrifuged for 10 min at 1500× *g* at 4 °C in order to obtain serum. After performing the routine diagnostic examination, the remaining part of serum was frozen and stored at −80 °C for further analysis.

### 2.6. Assesing the Serum Laminin Concentration

Laminin concentration was assessed using an enzyme-linked immunosorbent assay (ELISA) test supplied by Cloud-Clone Corporation (Katy, TX, USA). The microplate provided in the kit has been pre-coated with polyclonal anti-human laminin antibody. To each well sample/standard, biotin conjugated with anti-human laminin antibody, streptavidin–horseradish peroxidase (HRP) conjugate, substrate solution–tetramethylbenzidine (TMB) was added in exact order after proper incubation and washing between each step. The ongoing enzymatic reaction was stopped by the addition of acidic solution. The analytical sensitivity of the used method was determined as <3.2 ng/mL and the intra-run error was <10%.

### 2.7. Assesing the Serum Fibronectin Concentration

The concentration of fibronectin in the serum was determined using Human Fibronectin ELISA test from BioVendor Company (Brno, Czech Republic). To the wells pre-coated with polyclonal anti-human fibronectin antibody, samples/standards were added. Subsequently after proper incubation and washing, a biotin conjugated with anti-human fibronectin antibody was added. To induce and then stop the enzymatic reaction streptavidin-HRP conjugate, substrate (TMB) and acidic solution were added. The analytical sensitivity of the method was assessed as 0.1 ng/mL and the intra-run error was 5.3%.

### 2.8. Assesing the Serum Neutrophil Gelatinase-Associated Lipocalin Concentration

The NGAL concentration in the serum was assessed by the use of Human Lipocalin-2/NGAL ELISA test from BioVendor Company (Brno, Czech Republic). In this test samples and standards were incubated in the wells pre-coated with polyclonal anti-human lipocalin-2 antibody. Subsequently biotin labelled anti-human lipocalin-2 antibody, streptavidin-HRP conjugate and substrate solution (TMB) were added after incubation and washing following each step. The ongoing enzymatic reaction was stopped by the addition of acidic solution. The analytical sensitivity of used method was determined as 0.02 ng/mL and the intra-run error as 7.7%.

### 2.9. Statistical Analysis

The statistical analysis of the obtained data was performed with the use of STATISTICA software (version 13.3, StatSoft, Kraków, Poland). It involved verifying the normality of the data distribution using the Shapiro–Wilk test. Normally distributed variables were characterized by mean values and standard deviation (SD), while non-normally distributed variables were described using the median (Me) and the interquartile range including the lower (Q1) and the upper (Q3) quartiles. In order to verify the statistically significance of differences between the control group and the IBD group as well as the UC and CD groups, the Mann–Whitney U test in not normally distributed data and the Student’s *t*-test in normally distributed data were used. Furthermore, the sign test and the Student’s *t*-test were used to compare values of examined parameters before and after the treatment. In case of not normally distributed data, we conducted log-transformation prior to the Student’s t-test, meanwhile data not normally distributed even after log-transformation were analyzed by the sign test. For all conducted statistical analyses and tests, the significance level *p* < 0.05 was used.

## 3. Results

### 3.1. Research Data

The clinical characteristics of patients with UC and CD before and after a year of treatment have been summarized in Table 1 and Table 2. Data presented in Table 1 and Table 2 have already been published [8,9] as a part of our other research conducted on the same group of patients with IBD.

### 3.2. Quantative Changes of Serum LM, FN and NGAL in Patients with Ulcerative Colitis

The analysis showed that the concentration of FN and NGAL in the serum of patients with UC before treatment differed significantly from the concentration of these parameters in the serum of healthy subjects. Serum FN concentration in patients with UC before treatment decreased by 55% compared to the control group. At the same time, the concentration of NGAL in this UC group increased by 30% compared to healthy subjects. There was no significant difference between serum LM levels in patients with UC before treatment and in healthy individuals. Biological treatment had no effect on the plasma LM and NGAL profile in patients with UC. However, it caused significant changes in the concentration of FN in the blood serum. One year treatment with Adalimumab influenced the serum concentration of FN in patients with UC by increasing the concentration of the FN by the 35%.

### 3.3. Quantative Changes of Serum LM, FN and NGAL in Patients with Crohn’s Disease

**Figure 1:**
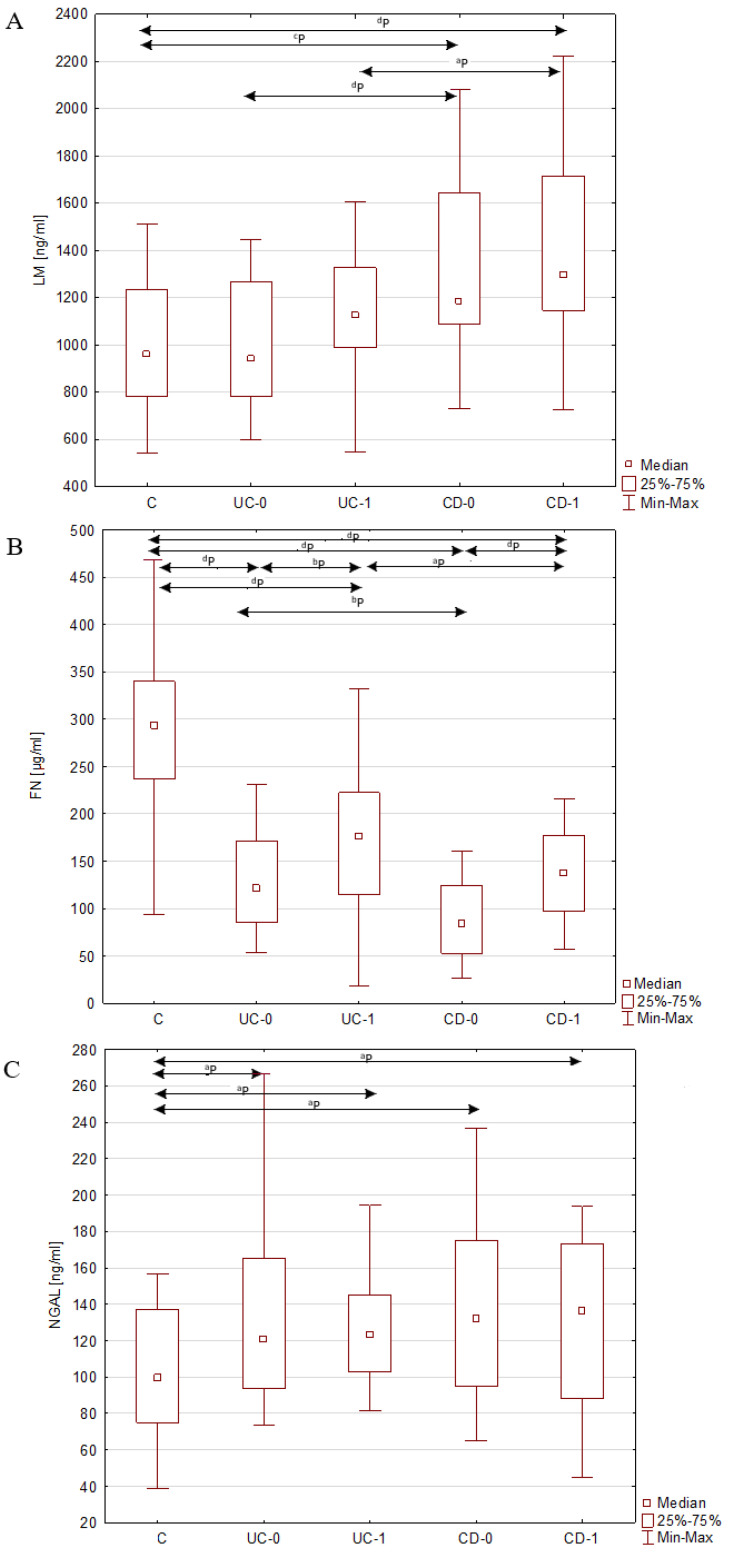
(**A**–**C**) Serum concentration of laminin (LM), fibronectin (FN) and neutrophil gelatinase-associated lipocalin (NGAL) in healthy individuals and patients with inflammatory bowel disease before and after a year of treatment. Presented results are expressed as median, interquartile range (25th–75th percentile), minimum and maximum of data in each group. CD-0, patients with Crohn’s disease before anti-inflammatory treatment; CD-1, patients with Crohn’s disease after a year of anti-inflammatory treatment; C, control; UC-0, patients with ulcerative colitis before Adalimumab treatment; UC-1, patients with ulcerative colitis after a year of Adalimumab treatment. ^a^ *p* < 0.05, ^b^ *p* < 0.01, ^c^ *p* < 0.005, ^d^ *p* < 0.001 statistically significant.

In the group of newly diagnosed patients with CD, the concentration of LM, FN and NGAL in the serum differed significantly from the concentration of these parameters in the serum of healthy individuals. Increase in concentration was noted for LM and NGAL and reached 31% and 35%, respectively. Serum FN concentration was reduced in pre-treatment patients with CD by 69% compared with blood FN concentration in healthy subjects. A similarly significant difference in the concentration of LM, FN and NGAL was noticed between the group of patients with CD after treatment and healthy people. The implemented one year anti-inflammatory treatment in patients with CD increased FN levels by 52%, but there was no significant difference in serum NGAL and LM levels before and after treatment. The comparison of the obtained results is presented in Table 3 and Table 4 and Figure 1.

### 3.4. The Relationship between Serum LM, FN, NGAL and Inflammatory Processes and Disease Activity

The studies revealed a significant relationship between the disease activity and the concentration of the analyzed parameters related to ECM in both major phenotypic forms of IBD, i.e., UC and CD. In the case of patients with UC, a significant correlation was noted between LM concentration and disease activity was assessed by the Mayo scale before (r = 0.35, *p* < 0.005) and after (r = 0.40, *p* < 0.005) a year of treatment with Adalimumab. Moreover, in patients with UC, a correlation between NGAL concentration and disease activity was observed, similarly, before (r = 0.49, *p* < 0.01) and after (r = 0.54, *p* < 0.005) Adalimumab treatment. On the other hand, in the group of patients with CD, a significant relationship was found only between LM concentration and disease activity assessed by CDAI, both before (r = 0.49, *p* <0.05) and after (r = 0.68, *p* < 0.005) years of anti-inflammatory treatment. The presented study also showed a positive effect of the implemented treatment in both groups of patients with IBD on the activity of the disease process. After one year of treatment, disease activity assessed by the CDAI or Mayo scale decreased significantly compared to the pre-treatment situation in patients with CD and UC, respectively.

The relationship between the blood concentration of ECM-related parameters and CRP used as a clinical marker of inflammation has also been evaluated. Statistical analysis revealed a significant correlation between the concentration of NGAL and CRP before (r = 0.56, *p* < 0.005) as well as after (r = 0.39, *p* < 0.05) a year of treatment in patients with UC. In the case of patients with CD, a significant relationship was observed only between the serum concentration of LM and CRP before anti-inflammatory treatment (r = 0.45, *p* < 0.05). A significant decrease in CRP values after treatment was noted only among patients diagnosed with UC. This subgroup of patients with IBD additionally presented decreased concentration of CRP compared to patients with CD after a year of treatment (*p* < 0.001).

## 4. Discussion

### 4.1. Quantative Changes of the ECM-Related Markers (LM, FN and NGAL) in the Serum of Patients with UC or CD before Therapy

The aim of our study was to investigate whether the remodeling of the extracellular matrix that occurs in the course of IBD is reflected in the quantitative changes in serum ECM-related markers. In the group of patients with UC before implementation of biological treatment, a significant difference has been noted between the concentration of FN and NGAL compared to the healthy individuals. In the case of patients with CD before implementation of anti-inflammatory therapy, the concentration of LM, FN and NGAL was significantly different from the values of these parameters in the serum of healthy subjects. The obtained results indicated that circulating profile of analyzed biochemical markers related to ECM and inflammation was significantly different in patients with IBD in comparison to healthy subjects. The data presented are consistent with the study of Yesil et al. [19] in which NGAL levels in the IBD group were increased compared to healthy individuals. Assessments of serum NGAL concentration made it possible to distinguish patients with IBD from healthy subjects with 76.1% sensitivity and 60.9% specificity (at a 129 ng/mL cut-off level of NGAL).

Under physiological conditions, the ECM is in a dynamic equilibrium between the synthesis and degradation of the components of the ECM. During inflammation, this balance is disturbed, among others due to the increased activity of MMPs. The binding of MMP-9 by NGAL protects the enzyme against TIMP inhibition, and thus contributes to the increased degradation of the intestinal ECM components, and further inflammation of the intestine. Activation of the pro-inflammatory NF-κB pathway in IBD by signaling through the Toll/IL-1 receptor contributes to the upregulation of NGAL at the site of inflammation. At the same time, the migration of granulocytes from the peripheral blood to the intestines observed in the course of IBD leads to an increased release of NGAL, which additionally acts as a chemoattractant to neutrophils and promotes their further migration to the inflamed tissue [2,4,6,17]. The above mechanism explains the increase in serum NGAL concentration in patients with UC and CD as compared to healthy subjects, noted in our studies. Our results are consistent with those reported by Oikonomou et al. [17] who noticed that the measurements of serum NGAL concentration can differentiate patients with IBD from healthy subjects or patients with irritable bowel syndrome.

With regard to laminin, our study reported a significant increase in its serum levels in patients with CD before treatment compared to healthy subjects. Observed increases might be related to the influence of pro-inflammatory cytokines, as they were found to increase laminin synthesis. In a study by Francoeur et al. [20], laminin synthesis was slightly increased by adding TNF-α or TGF-β to a normal human intestinal epithelial crypt cell line (HIEC), and the combination of TNF-α (stimulating inflammatory response via NF-κB pathway) with IFN-γ increased LM secretion, as well as its transcription level. These results indicate a significant influence of proinflammatory cytokines on the expression of basement membrane components [20,21]. Moreover, Koutroubakis et al. [22] analyzed the LM expression in patients with IBD and noticed that LM concentration was increased in patients with IBD compared to the healthy controls, which is consistent with the results obtained in our study.

Similar to laminins, fibronectins are also important adhesion glycoproteins among ECM macromolecules. Our investigation showed a significant difference in serum FN levels in both CD and UC patients compared to healthy subjects. FN is a glycoprotein involved in the healing process. In the early stage of intestinal healing, FN binds platelets and fibrin and then it is deposited in the wound bed [10]. Moreover, the accumulation of a matrix rich in fibronectin leads to further fibrosis of the extracellular matrix [11]. Allan et al. [14] noticed that plasma concentration of FN in patients with CD is decreased compared to healthy subjects and suggested that the lower FN level in the CD group may be caused by increased FN deposition. Similarly, Kochhar et al. [15] reported decreased plasma FN concentration in patients with UC compared to healthy individuals. The results obtained by Allan et al. [14] and Kochhar et al. [15] are consistent with those presented in this study, which confirms a significant role of fibronectin in the pathogenesis and progression of IBD.

### 4.2. The Influence of Implemented Treatment on the Profile of ECM-Related Markers in the Serum of Patients with IBD

The serum levels of FN in patients with IBD were dependent on the type of disease. Patients with UC had significantly higher blood levels of FN compared to those with CD, which may indicate the possibility of using the blood FN assessment as an indicator useful in the differential diagnosis of these two similar types of IBD. In addition, the applied therapy increased the concentration of FN in the blood of patients with IBD, both those with UC and those with CD. The mentioned difference may indicate that the introduced treatment decreased the accumulation of FN in the intestine and increased its release from the wound bed, which is observed in the final stage of healing. Therefore, the data presented in our study may indicate the healing of the mucosa taking place in patients with IBD during therapy, which is the main goal of IBD treatment.

The anti-inflammatory treatment implemented did not affect the concentration of LM in the serum of patients with CD and UC. However, patients with CD exhibited increased levels of LM compared to the UC group. Regarding the fact that the CRP levels were also higher in the CD group compared to UC group, the increase in LM concentrations observed may be related to the ongoing inflammation and increased activity of pro-inflammatory cytokines increasing the expression of laminins (TNF-α, IFN-γ) in intestinal epithelial cells. This thesis seems to be confirmed by the studies carried out by Komine-Aizawa et al. [23], which also showed an increase in TNF-α and IFN-γ in the serum of patients with CD compared to healthy controls. Thus, as with FN, the LM plasma profile was also clearly different between patients with CD and UC, which may be helpful in the differential diagnosis of these conditions.

### 4.3. The Influence of Inflammatory Processes and Disease Activity on ECM-Related Markers in Patients with IBD

Increased levels of CRP can help differentiate the mucosal activity in disease from quiescent IBD. Serum CRP as an acute phase reagent was found to decrease only in patients with UC after one year of treatment with Adalimumab. The results obtained are in line with the studies by Ogata et al. [24], who showed a significant reduction in CRP levels after 4, 24 and 52 weeks of adalimumab treatment. In the case of patients with CD, no significant decrease in CRP level was noted after a year of anti-inflammatory therapy in our study. Patients with CD presented increased values of CRP compared to patients with UC both before and after treatment. This observation is consistent with the results of the population-based study carried out by Henriksen et al. [25], where a higher level of CRP was noted among patients with CD compared to those with UC. Increased CRP levels in patients with CD may be related to more extensive tissue damage in this type of IBD, since lesions may involve all layers of the intestine, while in UC, they are limited to the submucosa.

The influence of inflammatory processes on the circulating level of the ECM-related protein was also investigated. Significant relationship was found between serum CRP and LM level in patients with CD before treatment. Moreover, a significant correlation was noted between the levels of CRP and NGAL in the serum of patients with UC, which indicates that NGAL may be a surrogate indicator of the intensity of inflammatory processes, helpful in monitoring the course of the disease. The decrease in NGAL concentration observed after adalimumab therapy may result from reduced migration of granulocytes to the diseased tissue, along with suppression of the inflammatory process [6]. On the other hand, it may be associated with inhibition of ECM degradation and damage of intestinal tissue, since NGAL protects MMP-9 from inactivation by TIMP-1. In the group of patients with UC, we did not identify any significant correlation between CRP levels and the disease activity scale. However, such correlation was noticed in the CD group. The difference observed may be related to the above-mentioned higher serum levels of CRP in patients with CD compared to those with UC.

The last part of the study concerned the assessment of the effect of disease activity on the plasma profile of ECM-related parameters in patients with IBD. Firstly, the studies showed that the applied therapy led to a reduction in the disease activity expressed in the Mayo scale in UC patients and the CDAI index in CD patients, respectively. These results are in line with other studies assessing the effectiveness of anti-TNF and corticosteroid treatment implemented in UC and CD patients [24,26].

Secondly, among the tested matrix glycoproteins, serum FN concentration did not correlate with disease activity; in the case of LM, a significant correlation was demonstrated with both the Mayo score in patients with UC and the CDAI index in patients with CD. LM was also the ECM-related parameter that differentiated patients with UC from those with CD. The results obtained indicate that the circulating LM can be used as a disease activity-dependent, non-invasive tool helpful in the differential diagnosis of these two similar types of IBD.

According to the research conducted, disease activity was also significantly correlated with the concentration of NGAL in the group of patients with UC. Similar results were presented in the study by Oikonomou et al. [17], where circulating NGAL levels in patients with active UC were increased compared with inactive disease. Oikonomou et al. [17] additionally noted a significant correlation between NGAL concentration and CDAI score in patients with CD. However, no such correlation was found in our study. Unfortunately, we were not able to assess faecal calprotectin levels in all patients in both study groups. The treatment regimens differed between the two research groups analyzed. In order to confirm our results regarding the role of ECM-related markers in the differential diagnosis between the two most common forms of IBD, similar treatment should be used in both groups of patients with IBD. Nevertheless, we believe that our findings make some contribution to the discovery of markers associated with ECM changes in IBD and may aid in clinical diagnosis and/or monitoring of the treatment progress. However, further studies should be carried out in groups of patients with a similar treatment regimen, as well as in a larger number of patients in order to confirm the results obtained.

## 5. Conclusions

In conclusion, the results indicate that the circulating profile of the ECM-related markers, including the most abundant non-collagenous proteins of the basement membrane such as laminin and fibronectin as well as serum level of neutrophil gelatinase-associated lipocalin, undergoes significant changes in IBD. These changes most likely reflect the remodeling of the intestinal extracellular matrix in both UC and CD groups of patients.

The analyzed ECM-related markers made it possible to distinguish patients with IBD from healthy individuals, and at the same time LM and FN measurements was also able to differentiate patients with UC from those with CD, presenting their usefulness as diagnostic markers. Moreover, the plasma LM profile in patients with CD and the concentration of LM and NGAL in the blood of patients with UC correlated with disease activity. Hence, the above-mentioned ECM-related markers can be used to monitor the effectiveness of implemented treatment and can be further considered as a non-invasive alternative for endoscopic examination.

## Figures and Tables

**Table 1:** Clinical characteristic of patients with ulcerative colitis (UC) before and after a year of treatment with Adalimumab.

Parameter	Patients with Ulcerative Colitis n = 31		*p*
	Before Treatment (UC0)	After Treatment (UC1)	UC0 vs. UC1
Mayo score	3 (2–3)	2 (1–3)	0.000 *
CRP (mg/L)	3.40 (1.26–17.51)	2.47 (1.51–7.68)	0.012 *
Glucose (mmol/L)	4.99 ± 0.7	4.81 ± 0.81	0.293
Cholesterol (mmol/L)	4.98 ± 0.8	4.93 ± 0.91	0.724
Triglycerides (mmol/L)	1.23 (0.87–1.36)	1.36 ± 0.47	**0.022**
Indirect bilirubin (μmol/L)	4.75 (1.8–7.7)	9.35 (5.5–16.35)	**0.000**
Direct bilirubin (μmol/L)	3.45 (1.9–3.8)	5.3 (3.6–8.2)	**0.000**
ALT (U/L)	15 (10–26)	16 (10–25)	0.809 *
AST(U/L)	17.92 ± 4.81	19 (15–23)	0.044 *
Creatinine (μmol/L)	79.88 (69–88.4)	74.7 (63.4–87.1)	0.137 *
Total protein (g/L)	73.48 ± 5.43	74.73 ± 5.63	0.231
Albumin (g/L)	42 (40–46)	43.34 ± 4.52	0.522 *
Sodium (mmol/L)	139.9 ± 1.93	140 (138–141)	0.190 *
Potassium (mmol/L)	4.17 ± 0.4	3.97 ± 0.33	**0.011**
Calcium (mmol/L)	2.36 ± 0.09	2.32 (2.25–2.44)	0.677 *
Hemoglobin (g/dL)	12.83 ± 2.28	13.5 ±2.3	**0.005**
Lymphocytes (%)	24.27 ± 10.71	28.3 (17.8–34.5)	**0.000**
Basophils (%)	0.76 ± 0.43	0.6 (0.45–1.1)	**0.000**
Eosinophils (%)	2.6 (1.1–3.3)	1.8 (0.8–2.6)	0.063
Monocytes (%)	5.72 ± 2.27	5.6 (4.5–7.8)	**0.000**
PLT (×10^9^/L)	372 (292–457)	342 ± 101,72	**0.000**

Data are presented as mean ± standard deviation (SD) or median and interquartile range (25th–75th percentile). Data have been analyzed with the use of Student’s *t*-test or sign test (*—data analyzed with the use of sign test); *p* < 0.05, statistically significant (bolded). ALT, alanine aminotransferase; AST, aspartate aminotransferase; CRP, C-reactive protein; PLT, platelets count.

**Table 2:** Clinical characteristic of patients with Crohn’s disease (CD) before and after a year of anti-inflammatory treatment.

Parameter	Patients with Crohn’s Disease *n* = 20		*p*
	Before Treatment (CD_0_)	After Treatment (CD_1_)	CD_0_ vs. CD_1_
Age (years)	32.1 ± 9.32		
CDAI	303.4 ± 52.45	273.85 ± 40.82	**0.017**
CRP (mg/L)	15.7 (4.2–39.05)	18.5 (9.4–28.75)	0.242
Sodium (mmol/L)	138.15 ± 2.89	138.38 ± 3.9	0.886
Potassium (mmol/L)	4.35 (4.2–4.45)	4.29 ± 0.22	0.606 *
Glucose (mmol/L)	4.94 (4.72–5.33)	4.89 ± 0.43	0.579 *
Creatinine (μmol/L)	81.33 ± 14.14	86.63 ± 13.26	0.300
WBC	7.2 ± 3.21	6.22 ± 1.97	0.630
RBC	4.04 (3.78–4.71)	4.25 ± 0.59	0.803 *
Hemoglobin (g/dL)	11.58 ±2.3	12.41 ± 1.94	0.411
HCT (%)	35.04 ± 5.54	37.09 ± 5.1	0.405
MCV (fL)	85.81 ± 8.44	86.21 ± 6.65	0.908
MCH (pg)	28.25 ± 3.55	29.7 (27.65–30.5)	0.803 *
MCHC (g/dL)	33.05 (31.45–34)	33.06 ± 1.57	0.803 *
RDWCV (%)	15.14 ± 2.35	14.01 ± 1.48	0.171
PLT (×10^9^/L)	364.68 ± 134.03	260.75 ± 118.45	**0.016**
PCT (%)	0.34 ± 0.12	0.27 ± 0.11	0.108
PLCR (%)	20.73 ± 5.23	10.28 ± 1.3	**0.000**
MPV (fL)	9.43 ± 0.72	11.55 (9.75–12.65)	**0.000**
PDW (fL)	10.3 ± 1.26	11.55 (9.75–12.65)	**0.000**

Data are presented as mean ± standard deviation (SD) or median and interquartile range (25th–75th percentile). Data have been analyzed with the use of Student’s *t*-test or sign test (*—data analyzed with the use of sign test); *p* < 0.05, statistically significant (bolded); CDAI, Crohn’s Disease Activity Index; CRP, C-reactive protein; HCT, hematocrit; MCH, mean corpuscular hemoglobin; MCHC, mean corpuscular hemoglobin concentration; MCV, mean corpuscular volume; MPV, mean platelet volume; PCT, plateletcrit; PDW. Platelet distribution width; PLCR, platelet-large cell ratio; PLT, platelet count; RBC, red blood cells; RDWCV, red blood cell distribution width, coefficient of variation; WBC, white blood cells.

**Table 3:** Comparison of serum laminin (LM), fibronectin (FN) and neutrophil gelatinase-associated lipocalin (NGAL) concentrations in patients with UC and CD at baseline and after a year of treatment.

Parameter	Control *n* = 48	Patients with Ulcerative Colitis *n* = 31		Patients with Crohn’s Disease *n* = 20	
		Before Treatment (UC_0_)	After Treatment (UC_1_)	Before Treatment (CD_0_)	After Treatment (CD_1_)
LM [ng/mL]	1012.07 ± 260.85 CI (217.15–326.74)	1016.40 ± 259.40 CI (386.38–658.49)	1138.05 ± 273.77 CI (285.22–486.08)	1329.50 ± 389.36 CI (296.10–568.68)	1400 ± 412.56 CI (313.75–602.58)
FN [μg/mL]	287.93 ± 79.69 CI (66.34–99.82)	130.56 ± 52.87 CI (42.25–70.67)	176.88 ± 82.02 CI (65.54–109.63)	89.26 ± 43.86 CI (33.35–64.06)	135.89 ± 49.86 CI (37.92–72.82)
NGAL [ng/mL]	102.65 ± 37.39 CI (28.43–54.60)	133.34 ± 51.51 CI (41.16–68.85)	125.80 ± 28.37 CI (22.67–37.92)	138.94 ± 51.31 CI (39.02–74.94)	131.10 ± 46.39 CI (35.28–67.75)

Data are presented as mean ± standard deviation (SD). FN, fibronectin; LM, laminin; NGAL, neutrophil gelatinase-associated lipocalin. CI—confidence interval.

**Table 4:** Statistical significance of changes in serum laminin (LM), fibronectin (FN) and neutrophil gelatinase-associated lipocalin (NGAL) concentrations between analyzed groups.

Parameter	Groups	*p* Value
**LM**	C vs. CD_0_	0.002
	C vs. CD_1_	0.000
	UC_0_ vs. CD_0_	0.000 *
	UC_1_ vs. CD_1_	0.018
**FN**	C vs. CD_0_	0.000
	C vs. CD_1_	0.000
	C vs. UC_0_	0.000
	C vs. UC_1_	0.000
	UC_0_ vs. UC_1_	0.005
	CD_0_ vs. CD_1_	0.000
	UC_0_ vs. CD_0_	0.005
	UC_1_ vs. CD_1_	0.031
**NGAL**	C vs. UC_0_	0.017
	C vs. UC_1_	0.015
	C vs. CD_0_	0.015
	C vs. CD_1_	0.04

Presented data include only *p* values with statistical significance. Data were analyzed by the use of the Mann–Whitney U test (*) in not normally distributed data or by the Student’s *t*-test in normally distributed data; CD_0_, patients with Crohn’s disease before prednisone treatment; CD_1_, patients with Crohn’s disease after a year of anti-inflammatory treatment; C, control; UC_0_, patients with ulcerative colitis before Adalimumab treatment; UC_1_, patients with ulcerative colitis after a year of Adalimumab treatment.

## Data Availability

Data are contained within the article.
